# A Brief Overview on Crack Patterns, Repair and Strengthening of Historical Masonry Structures

**DOI:** 10.3390/ma16051882

**Published:** 2023-02-24

**Authors:** Reza Latifi, Marijana Hadzima-Nyarko, Dorin Radu, Rahimeh Rouhi

**Affiliations:** 1 School of Civil Engineering, University of Bologna, 40126 Bologna, Italy; 2 Faculty of Civil Engineering and Architecture Osijek, Josip Juraj Strossmayer University of Osijek, 31000 Osijek, Croatia; 3 Faculty of Civil Engineering, Transilvania University of Brașov, 500036 Brasov, Romania; 4 Department of Radiation Oncology, Gustave Roussy Cancer Campus, 94800 Villejuif, France; 5 Université Paris-Saclay, Institut Gustave Roussy, Inserm, Radiothérapie Moléculaire et Innovation Thérapeutique, 94800 Villejuif, France

**Keywords:** masonry walls, vault, crack patterns, strengthening techniques

## Abstract

Given that a significant fraction of buildings and architectural heritage in Europe’s historical centers are masonry structures, the selection of proper diagnosis, technological surveys, non-destructive testing, and interpretations of crack and decay patterns is paramount for a risk assessment of possible damage. Identifying the possible crack patterns, discontinuities, and associated brittle failure mechanisms within unreinforced masonry under seismic and gravity actions allows for reliable retrofitting interventions. Traditional and modern materials and strengthening techniques create a wide range of compatible, removable, and sustainable conservation strategies. Steel/timber tie-rods are mainly used to support the horizontal thrust of arches, vaults, and roofs and are particularly suitable for better connecting structural elements, e.g., masonry walls and floors. Composite reinforcing systems using carbon, glass fibers, and thin mortar layers can improve tensile resistance, ultimate strength, and displacement capacity to avoid brittle shear failures. This study overviews masonry structural diagnostics and compares traditional and advanced strengthening techniques of masonry walls, arches, vaults, and columns. Several research results in automatic surface crack detection for unreinforced masonry (URM) walls are presented considering crack detection based on machine learning and deep learning algorithms. In addition, the kinematic and static principles of Limit Analysis within the rigid no-tension model framework are presented. The manuscript sets a practical perspective, providing an inclusive list of papers describing the essential latest research in this field; thus, this paper is useful for researchers and practitioners in masonry structures.

## 1. Introduction

Masonry buildings are massively used by more than one-third of the world’s population compared to reinforced concrete and steel structures due to local availability, recyclability, low-cost sustainable construction work, relatively good thermal behavior, and acoustic insulation qualities [1]. Recent seismic events have highlighted that masonry buildings are structurally vulnerable and characterized by fragile and sudden failures, e.g., the Bam (Iran) earthquake in 2003, the L’Aquila (Italy) earthquake in 2009 [2], or Durres (Albania) in 2019 [3].

Medieval and gothic architecture, especially cathedrals, are widespread all over Europe and are an inherent part of the unique heritage to be preserved. Several architectural innovations, particularly in church construction, were developed during the Romanesque and Gothic periods. The main Romanesque characteristics are round arches, perimeter walls with small windows, barrel vaults, large pillars, and large walls. The pointed or ogival arch, as well as ribbed cross vaults, flying buttresses, large openings, rose windows, tie rods, and slender columns, characterize gothic architecture. Gothic cathedrals have relatively slender piers; thus, considering the need to reduce the buckling length, but also to counteract the thrust of the arches and/or vaults, the piers and the vaults are attached to flying buttresses, structural elements commonly that have evolved in the Gothic era from earlier, simpler, to hidden supports. The design increased the supporting power of the buttress and allowed for the creation of the high-ceilinged churches typical of Gothic architecture. One of the examples is San Petronio cathedral, one of the most prominent Gothic architectural structures in Bologna, and it is 132 m in length, 66 m in width, and 47 m in height, with a chapel, chancel, cloister, aisle, apse, nave, and sacristy spaces. Other examples worth mentioning are the Gothic-style church San Martino Maggiore, the San Francesco church, and the Garisenda leaning tower from Bologna. The structural solution for these historical buildings is usually unreinforced masonry. Several elements are used for stabilizing the structure against lateral loadings. The buttress is a structural element built near a wall to transfer the vaulted ceiling’s lateral thrust forces to the ground to carry wind or earthquake loading. The flying and the side buttresses provide support against the lateral and horizontal vault or arch thrusts and lateral loading. The flying buttress with fixed ends is nondeformable, and its stability depends only on the masonry crushing strength of the arch support. The side buttresses are stocky because their weight must balance the thrust action and provide extra vertical loading to support the wall or resist lateral thrust transmitted by the arch. The buttresses and drum may be extremely weak to contribute to static stability and support the dome with a heavy lantern, so iron hoops can be installed to encircle the dome, balancing the drum and buttresses’ static deficiency. Several studies are presented in the literature that describe the role and evolution of the buttress as a supporting structural element to URM walls. Dimitri et al. [4] present a numerical study on the dynamic behaviour of masonry arches with buttresses, highlighting the failure and collapse modes of the URM structures under horizontal ground motions using the discrete element method. Additionally, Ochsendorf et al. [5] investigated the collapse of the masonry buttresses under concentrated lateral loads using the Heyman masonry model [6]. A different approach was used in a study by Liu et al. [7] in which the URM behavior with different opening positions is revealed, following push over analysis.

Considering the role of flying buttresses in gothic architecture, studies can be found in the literature that present flying buttresses’ behaviour in different approaches. A substantial analysis was conducted by Nikolinakou and Ochsendorf [8] in which they detail the influence of the thrust line position and dimensions of the flying buttress arch for early gothic structures. Additionally, Datoussaïd et al. [9] studied the behaviour of flying buttresses in the case of the Turnai (Belgium) gothic cathedral, using the finite element method.

There are studies evaluating the thrust and stresses in the piers for existing historical Renaissance architectural structures. One of the examples is a new study regarding Brunelleschi’s Dome [10] from Florence city, which presents a kinematical approach in the context of the Heyman masonry model [7]. Comparisons are made with other valuations made by the usual but less accurate statical approach. The knowledge of the thrust allows an evaluation of the stresses acting in the supporting piers: their base sections are all compressed, with level stresses sufficiently low.

A Gothic-style pointed or ogival arch can support heavy loads, allow more open space, and produce less thrust at the base than a round arch. The flat arches are composed of voussoirs that efficiently use the masonry’s compressive strength and should be adequately buttressed on either side to resist lateral thrust and partial collapse.

The usual unreinforced masonry structures are especially sensitive to lateral loads, like the shear forces associated with wind and/or seismic loads [11,12,13]. Therefore, most exhibit different behaviors due to the lack of reinforcement to resist the tensile stresses induced by lateral loading [14,15]. Furthermore, special considerations should be made in slender, more vulnerable structures such as church bell towers [16,17]. Masonry high-rise structures such as bell towers are more vulnerable to lateral loads, e.g., wind and earthquakes, which are random vibrations. Wind loads repeatedly affected the masonry bell towers, leading to resonance phenomena and crack propagation. Several studies present the effect of the bell ringing on historical masonry structures [18]. Vicenzi et al. present a dynamic monitoring and structural assessment of the masonry bell tower [19] by means of a structural integrity assessment and monitoring of the stress values following bell-induced displacements.

Nowadays, with the advancement in technological solutions and novel materials, engineers are offered a wide range of retrofit and consolidation options as alternatives to preserve every structure, such as adding new structural elements, strengthening existing elements, locally increasing the deformation capacity, seismic isolation, demands reduction, etc. [20]. An intervention strategy is obtained by decreasing the loads rather than increasing the capacity, inserting new structural elements, or strengthening some elements to reduce brittle failure modes and increase system displacement capacity. In the last decade, composite reinforcing systems have been practiced for static or seismic retrofitting masonry walls, arches, vaults, and piers [21].

Regarding the (URM) walls, considering the local failure modes [22] for the masonry walls subjected to in-plane forces, basically, three failure mechanisms can be identified following a visual inspection: the one associated with the brittleness of the material (which manifests with detachment-type fractures), the second which is due to in-plane shear (usually in walls subjected to compression and shear loads), and the third in which the structure is damaged following the high compression values consisting of detachment fractures with damaged material [23]. Considering an in-depth analysis of the URM walls regarding the failure modes, other typical failure modes can be identified that are related to tension, compression, and shear forces. Thus, sliding following shear or diagonal shear is another failure mode of the unreinforced masonry walls. Regarding this matter, several studies are presented in the literature. Thus, Najif and Khattak [24] present the observed failure modes following the 2015 Hindu Kush earthquake, concluding that partial or complete out-of-plane collapse of the URM walls was due to a lack of shear resistance and flexural cracking in the spandrels. Research results adopt the 2017 ASCE 41 standard for Seismic Evaluation and Retrofit of Existing Buildings. More recently, in 2021, Casapulla [25] evaluated the seismic response of out-of-plane loaded URM walls by means of nonlinear analysis, investigating the out-of-plane failure modes for masonry buildings vulnerable to seismic loads.

Essentially, the behavior of masonry structures is a structural integrity issue, and the literature is abundant in studies regarding crack patterns in masonry structure walls. Several studies should be pointed out, such as from Korswagen et al. [26], who developed a study for masonry walls under shallow earthquake loadings in unreinforced masonry (URM) walls employing fracture mechanics, or Bamonte and Taliercio et al. [27], who propose a finite element modeling of cracks induced in a masonry chimney. The work of Varale-Rivera et al. is also worth mentioning, considering that their research studied crack patterns and proposed different retrofitting solutions for URM walls under earthquake loading.

The unreinforced masonry foundations transmit vertical loads (usually vertical loads) to the soil by direct bearing, and their retrofit includes injection grouting, reinforcing, prestressing, and enlargement. According to the latest research and standards, the most reliable retrofit strategies require judgment on a case-by-case basis; therefore, the present research aims to discuss mechanical testing, masonry damage/crack patterns, possible failure mechanisms, and traditional and innovative strengthening solutions. For example, steel or timber tie-rods are mainly used to support the horizontal thrust of arches, vaults, and roofs. Composite reinforcing systems can improve tensile resistance, ultimate strength, and the displacement capacity of strengthened masonry. In addition, the kinematic and static principles of Limit Analysis within the rigid no-tension model domain for masonry structures are illustrated.

The primary standards for developing guidance on evaluating and repairing masonry walls and the damage effects on stiffness, strength, and displacements for masonry components are FEMA 306, 307, and 308 [28].

There are several solutions to be presented in this paper regarding the retrofitting of masonry walls—from steel-reinforced grout (SRG) or fabric-reinforced cementitious matrix (FRCM) to additional structural elements as consolidation solutions.

The steel-reinforced grout (SRG) and fabric-reinforced cementitious matrix (FRCM) composites have principal advantages in retrofitting masonry structures: low weight, high tensile strength, large deformation capacity, crack propagations, compatibility with the masonry substrate’s physical and mechanical features, corrosion resistance, and removability.

The mortar matrix preserves the embedded fabric consisting of yarn grids made of carbon, glass, and aramid, functioning in stress transfer among the fibers and masonry substrate [29]. The mortar comprises fine-grained aggregate, cement, micro silica, pozzolan, natural hydraulic lime, discontinuous short fibers, and polymeric additives. The microfibers are incredibly efficient in enhancing mortar shrinkage resistance and reducing plastic shrinkage. Alkali-resistant glass fabrics have long-term durability with cementitious matrixes, resisting degradation and aging due to environmental corrosion in different temperatures. The carbon fibers present excellent chemical resistance against moisture attack, high temperatures, freezing-and-thawing cycles, and an alkaline environment [30]. Mortar penetration within the fabric holes may improve the mechanical properties depending on mortar matrix viscosity and fabric network [31]. The architectural heritage preservation requirements comply with lime-based mortars: chemical and mechanical compatibility with masonry substrates, vapor permeability, removability with no substrate damage, and reversibility.

The increase in shear and flexural capacity of externally bonded FRCM/SRG to masonry depends on the unidirectional or bidirectional fiber’s tensile stress, while the masonry structural element carries the compressive stress the same as RC. Therefore, the intervention effectiveness depends on the bonding features at the composite-to-masonry interface, mortar joints, and surface roughness. The bond performance between embedded fibers in matrix and masonry substrates is critical due to the possible brittle adhesion failure mechanism or debonding [32]. The probable debonding failure modes include a failure in the substrate, composite-substrate interface, fabric matrix interface, fabric-mortar slippage, and fabric tensile rupture. The substrate cohesive debonding may happen if a relatively strong matrix is bonded to an almost weak substrate. The substrate matrix interface detachment occurs on relatively smooth surfaces.

## 2. Methods for Testing the Masonry Structural Elements

Masonry structures are challenging to assess due to the heterogeneity of materials and their mechanical behavior. Much research attention has been paid to monitoring their structural health. In many recent publications, new advanced technological methods have been provided, such as cheaper sensors, wireless connections, non-contact surveys, and continuous monitoring.

A comprehensive interdisciplinary science can diagnose a masonry building based on technological surveys, non-destructive testing (NDT), destructive testing (DT), and the interpretation of crack and decay patterns. The damage causes, crack patterns, deficiencies, and material characteristics should be identified and addressed before performing repairs. The tensile strength of fired bricks, stone blocks, and mortars is expected to be approximately 1/10 to 1/15 of compression strength representing brittle behavior [2].

The standard for practicing ultrasonic techniques for masonry testing has been developed in Europe by the RILEM Committee, TC 127-MS (2001) [33]. The impact-echo method can be helpful for determining the location of void areas in grouted reinforced masonry (RM) walls, as described in [34] in 1997.

DTs are generally not conducted in architectural heritages due to regularly expensive instrumentation and execution time, e.g., diagonal compression test, single and double flat jack test, double punch test, and Brazilian test with a rotated mortar joint penetrometer test [35]. The expected shear strength of URM elements is estimated from bed-joint shear strength measurements utilizing the in situ shear test described in ASTM C1531 or tensile splitting tests as prescribed in ASTM C496 or diagonal compression (shear) experiment based on ASTM E519. According to ASTM E519, masonry panels with different mortar bed strengths are subjected to a diagonal compression force to the brickworks to check the masonry’s shear capacity. URM walls’ existing vertical compressive stress, deformability properties, and elastic modulus are estimated using the flat jack method according to ASTM C1196, ASTM C1197, and TMS 402/602-16 [36].

Several NDT techniques are generally based on propagating and detecting the material’s ultrasonic pulses and electromagnetic waves, such as impact-echo and infrared thermography tests that exhibit material density variations, homogeneity, cracks, and debonding. According to [37], the test equipment with wave frequencies in the 50 kHz range is appropriate for the masonry wall assessment. Higher frequencies are not recommended since short wavelengths are inconsistent with the typical masonry unit dimensions. The radiographic and infrared thermography instruments can identify the reinforcing steel location, conduits, pipes, and chimneys in masonry walls.

The surface penetrating radar method involves transmitting high-frequency microwave electromagnetic radio pulses into the material, measuring the time elapsed between transmission to detect voids and other defects in multi-wythe masonry walls, and assessing the injection repairs’ effectiveness. The Schmidt rebound hammer is used to estimate the surface hardness of exterior masonry, and the linear variable differential transformer (LVDT) is applied in crack widening measurements.

Destructive testing (DTs) on FRCM/SRG composites such as pull-off, pullout, direct tensile, single-lap shear tests are examined for matrix tensile strength, in order to determine the ability to bond to the substrate, and slippage [38]. In a single-lap shear test to draw an axial stress-slip relationship, a specimen is restrained by an endplate while the unbonded fabric end is clamped and pulled out, and transducers measure the slip or relative displacement of fabric and substrate. Various failure modes may occur in this test, such as debonding by substrate cohesive failure, matrix-to-substrate detachment, fabric-to-matrix interface detachment, fabric slippage within the mortar matrix, and fabric tensile rupture [39]. The substrate crushing failure mode may occur when high fabric reinforcement ratios are applied, and ultimate capacity is governed by masonry compressive strength [40].

As early as 2008, Tung et al. [41] proposed a Digital Image Correlation (DIC) method to investigate masonry walls. In 2021, Howlader and Griffith [42] proposed several tests for URM walls under in-plane cyclic loading and constant vertical pre-compression load, measuring the displacements and assessing the crack patterns utilizing DIC. The tests’ conclusions are relevant to the literature, understanding the overall behavior of the masonry walls at cyclic lateral loading (e.g., seismic type loading) through the structural integrity approach. Similar tests were conducted by Torre et al. [43], who compared the results of the tests with the retrofitted structural URM walls with a Fiber-Reinforced Cementitious Matrix (FRCM) employing DIC. The results were calibrated with a linear variable differential transformer (LVDT), thus confirming the research results. As a conclusion of these studies, the DIC procedure can be used as an NDT for URM-type structure elements in experiments and on-site tests.

Other NDT methods, like Structural Health Monitoring (SHM) or Condition Monitoring (CM), are presented in recent studies like that from Pallarés et al. [44] for determining structural health assessments through signal processing tools validated by numerical analysis.

### Automatic Surface Crack Detection

In this section, we introduce Machine Learning (ML) and Deep Learning (DL), then review some state-of-the-art works on crack detection based on ML and DL. ML is one of the applications of Artificial Intelligence (AI). ML aims to develop algorithms that can automatically recognize patterns in data and subsequently use the patterns to predict unseen data [45]. DL is a part of ML, which designs the complex structure of algorithms in layers to create artificial neural networks, modeled on the human brain, that are capable of learning from input and labeled data to predict unseen data [46]. ML and DL’s general pipeline includes preprocessing, segmentation, feature extraction and selection, and classification. The applied number of stages in the pipeline depends on the problem and its application. We give some explanation on each stage as follows:

Preprocessing: Images in a dataset may come from various sources, having different characteristics like type and size. To feed the images into an ML or a DL model, preprocessing is needed to harmonize all the applied images that have undergone the same analysis. Preprocessing usually includes resizing, cropping, normalization, contrast enhancement, etc.

Segmentation: This refers to partitioning an image into different regions or image objects, more generally, Region Of Interests (ROIs).

Feature extraction: When the input data, e.g., a set of images, is too large to be processed by an algorithm, it can be transformed into a reduced set of features that characterize samples from different classes.

Feature selection: This stands for choosing a subset of extracted features so that the feature space is optimal. In other words, the most critical features, which effectively separate samples of different classes according to specific criteria, are selected.

Classification: Also known as supervised learning, this is the task of categorizing a given set of data into a specific number of classes based on the selected features.

Each ML/DL model needs training, validation, and test datasets. Training data is a subset of the data on which the model training and optimization are iteratively performed in some approaches. Validation data improve the model performance by fine-tuning the model parameters for optimization after each epoch. The test data are applied to measure the accuracy of the trained model on unseen data.

There are two ways of splitting data for ML/DL models. The first one is called standard division, through which the dataset is divided into 70%, 15%, and 15% for model training, validation, and testing. Cross-validation is another technique used to overcome overfitting, which is defined as memorizing data by an ML/DL model instead of learning from data by training the model on different subsets of data. Based on 10-fold cross-validation, as an example, the dataset is divided into 10 folds; 9 folds are applied in training, and the remaining 1 fold is used for the validation of the model. It is repeated 10 times by swapping the training and the validation folds. In doing so, all the single folds are considered in the validation. To have a benchmark and better understanding of the performance of different ML/DL models, several metrics such as precision, recall, accuracy, and so on are calculated. The average values obtained from the computation of each of these metrics on the samples in the single folds in 10-time repetition of cross-validation is considered the final evaluation of the trained models.

Over the last decades, ML and DL have been increasingly applied, providing faster and more accurate systems by automating tasks such as crack detection. We review some of the recent works for crack detection with different applications as follows:

In 2022, Elhariri et al. [47] proposed a crack detection method in images of historical buildings by extracting three types of feature sets, including hand-crafted features, Convolutional Neural Network (CNN) learned features, and a fusion of hand-crafted and CNN-learned features. Their method was validated by implementing several classifiers based on three-fold cross-validation. Two datasets of crack images were used for developing the feature sets. Their results show that both Support Vector Machine (SVM) and stacked ensemble classifiers achieved the highest accuracy of 98% for crack detection using the CNN-learned features with dimensionality reduction.

Additionally, Gehri et al. [48] presented a fully automated procedure to detect cracks and measure crack kinematics in laboratory experiments instrumented with digital image correlation (DIC). They extracted crack lines using well-established image processing methods, which showed excellent agreement with the physical crack pattern. Instead of using pixel intensities of non-crack images, they used the DIC principal tensile strain field to extract much finer cracks and more reliable crack locations. More specifically, the crack widths and slips were measured using the DIC displacement field, accounting for local rotations of the specimen. They also presented automated visualizations of the crack kinematic measurements, including data smoothing. They did several sensitivity analyses by evaluating the crack detector’s performance and uncertainty and the crack kinematic measurements. With appropriate DIC parameters, their proposed method detected crack locations with high precision and measured crack kinematics very accurately, even in large-scale experiments with complex crack patterns.

Fan et al. [49] developed a fully automatic crack detection based on DL, particularly an encoder-decoder architecture with hierarchical feature learning and dilated convolution, named U-Hierarchical Dilated Network (U-HDN). Crack characteristics with multiple context information were automatically able to learn and perform end-to-end crack detection. They applied a public crack database including 118 images resulting in better methods on the same images. Their proposed U-HDN method achieved high performance because it extracted and fused different context sizes and levels of feature maps compared to other algorithms.

Chen et al. [50] proposed an automatic crack detection method that fused 3D point clouds and 2D images based on an improved Otsu algorithm. Initially, a high-precision registration of a depth image projected from 3D point clouds and 2D images was implemented. Then, pixel-level image fusion was performed, which fused the depth and gray information. Next, a rough crack image was obtained from the fusion image. Finally, the connected domain labeling and morphological methods were used to extract the cracks finely. They reported an average precision of 89.0%, recall of 84.8%, and F1 score of 86.7%, performing significantly better than the single image (average F1 score of 67.6%) and single point cloud (average F1 score of 76.0%) methods.

## 3. Rigid No-Tension Model, Static and Kinematic Principle

**Figure 1:**
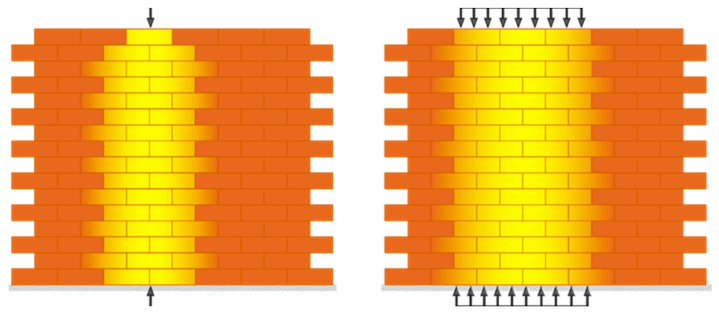
Weak dispersion of point and distributed loads within masonry walls.

The Limit Analysis initially formulated in the late 20th century for ductile steel structures can also be utilized for masonry structures satisfying the no-tension assumptions. In 1966, Jacques Heyman formulated constitutive assumptions in determining the admissibility domain of the no-tension, rigid in compression masonry models: (1) masonry is incapable of withstanding tensions, or the masonry has no tensile strength; (2) the infinite compressive strength of masonry; (3) negligible elastic strains; and (4) sliding cannot happen since masonry has infinite shear strength and the only possible deformation is detachments or cracks [51]. The admissible domain or stress tensor of the masonry continuum defines a significant consequence: the loads do not scatter or diffuse within a masonry continuum. However, according to the St. Venant principle, linear elastic bodies’ behavior is different, spreading the point or distributed loads action. As described within the no-tension framework, the transmission of vertical points and distributed loads in a masonry wall are approved by masonry bulk composed of bricks and mortar beds, illustrated in Figure 1. The stress evaluation within masonry vertical wall bands is significantly related to bricks and mortar bed characteristics.

The fundamental theorems of Limit Analysis (LA) can be stated in static or lower bound theorem, and the kinematic or upper bound theorem and Heyman’s research highlight that the Limit Analysis allows for considering the masonry structure in the ultimate state.

The mechanism of a masonry macro element is due to the opening of fractures that separate the macro element into rigid blocks. In the following, the kinematic and static theorems of Limit Analysis are used in their unique form for masonry constructions. Equation (1) represents the Principle of Virtual Work (PVW) for masonry:

(1) $$\langle \sigma ,\;\delta \varepsilon \rangle =\langle r,\;\delta u\rangle +\langle p,\;\delta u\rangle +\left\{t^{\left(n+\right)},\;{∆}^{\left(n-\right)}\delta u\right\}$$

where 〈σ, δε〉=0: work of internal stresses (σ) on the strains/deformations (δε) within the masonry bulk, which is zero; 〈r, δu〉: work of reactions (r) on corresponding displacement fields (δu); 〈p, δu〉=〈g+λq, δu〉: work of permanent (g) and live loads (λq) on corresponding displacement fields (δu); $\left\{t^{\left({\hat{n}}^{+}\right)},\;{∆}^{\left({\hat{n}}^{-}\right)}\delta u\right\}$: work of traction vectors ($t^{\left(n\right)})$ concerning the plane defined by the outward-normal vector $\hat{n}$ on relative displacement fields $\left({∆}^{\left({\hat{n}}^{-}\right)}\delta u\right)$ that must be perpendicular to the crack.

Studying the minimum arc thrust utilizing the static or kinematic theorem enables determining where the hinges have formed and the safety factor in the current state. If the masonry structure is subjected to permanent load *g* and live loads $\lambda^{-}q$, where $\lambda^{-}$ is the multiplier of load *q*, the static theorem states that loads $g+\lambda^{-}q$ are not greater than the collapse load $g+\lambda_{c}q$ if an equilibrium exists between the loads and internal stresses. The kinematic theorem states that if a structure is assumed in a mechanism state under loads $g+\lambda^{+}q$, the kinematic multiplier $\lambda^{+}$ cannot be lower than the collapse multiplier $\lambda_{c}$. The assumed loading condition for the arch subjected to horizontal forces is that the arch weight remains constant while the horizontal forces increase by λ multiplier. The arch’s load-bearing capacity is increased if the arch is reinforced at either intrados or extrados with composite materials; therefore, kinematic hinge mechanisms are not free to develop [52].

**Figure 2:**
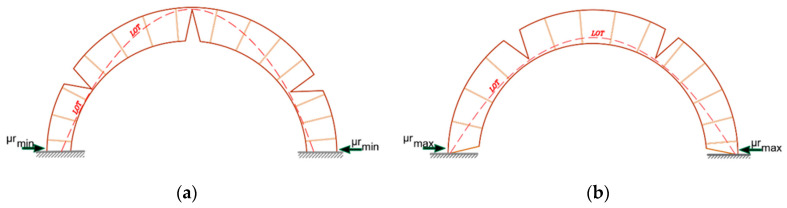
Settlement state: (**a**) minimum horizontal thrust; (**b**) maximum horizontal thrust.

Hence, a minimum thrust state occurs in the arch or vault if piers or supports retain the thrust experience settling, leading to a slight widening of the web spans and cracks. As long as the line of thrust (LOT) lies within the thickness, mechanism configurations are stable. Figure 2 shows a settlement state for an entire semicircular arch, assuming the mechanism defined by the hinge positions. Scenario (a) corresponds to the case of minimum thrust in which hinges form away from the abutments and the abutments move outward. Whereas scenario (b) represents the maximum thrust, in which the abutments moved slightly closer. As defined in equation (2), the kinematical approach will estimate the minimum horizontal thrust $\mu r_{min}$ of the settled arch that can be achieved as the maximum of all the kinematical thrusts μr defined by virtual work equation utilization:

(2) $$\mu_{s}=max\left\{-\frac{\langle p,\;\delta u\rangle }{\langle r,\;\delta u\rangle }\right\}=max\left\{-\frac{\langle g,\;\delta u\rangle \;}{\langle r,\;\delta u\rangle }\right\}$$

where 〈g, δu〉 is work of dead loads/weights g acting on the arch on mechanism δu, which is positive, and 〈r, δu〉 is resisting the work of thrust $\mu_{s}r$ on the horizontal settlement mechanism of the arc δu, which is negative.

### Arch, Vault, and dome Statics

Leonardo Da Vinci initially formulated the arch definition using an inverted catenary and introduced a practical method to evaluate the arch thrust using hanging weights and pulleys. The arc is produced by an inverted catenary that can support identical loads in compression instead of tension. The LOT is a theoretical line representing the compressive forces’ resultant path within the arch, and since the arc is statically indeterminate, there is an infinite number of LOT. The arch is safe as long as the LOT lies entirely within the arch, and a mechanism may develop if the LOT touches the arch intrados (interior surface) or extrados in at least four points. Couplet (1731) estimates the minimum admissible thickness of a round arc with the formula (t/r)_min_ = 0.108, in which t is the thickness and r is the internal radius [52].

**Figure 3:**
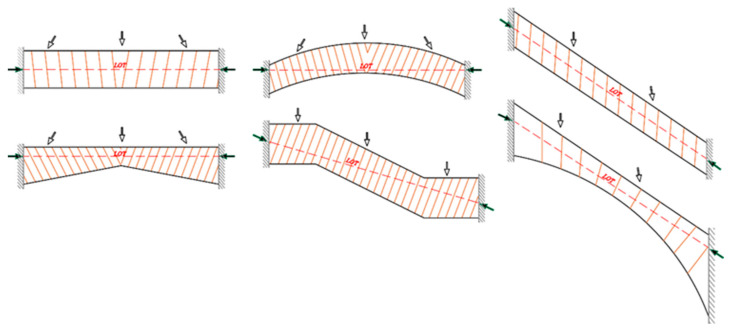
Non-deformable arches.

Some masonry structural systems cannot be deformed to develop mechanisms due to geometries and constraints since material interpenetration arises for any hinge position, like a stair ramp, flying buttress, or steel plates installed between fixed constraints, as illustrated in Figure 3. This status is a consequence of the compressional rigid material hypothesis. The rigid stones or brick impenetrability requires that the relative displacement among two points across two crack edges occur perpendicularly to the crack. The masonry nondeformable systems, e.g., flying buttress, flat arc, and stair ramp, cannot be deformed to form mechanisms due to their constraints and geometries, and the LOT is always within their thickness. The material interpenetration strength and fixed restraints limit any deformation, and only compressions may occur in the masonry bulk. So, nondeformable elements can always support the applied loads without forming any collapse mechanisms, except constraints are relocated by settling or material experiences crushing.

**Figure 4:**
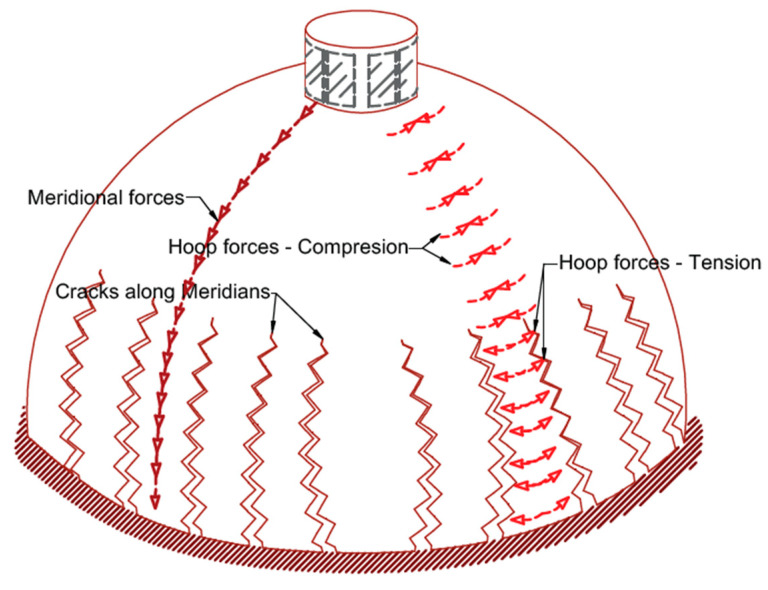
The behavior of a spherical dome under gravity loads.

Figure 4 schematically illustrates a spherical dome under gravity loads with meridian and hoop forces. The primary stresses occurring in a dome before cracking are characterized by the membrane solution, which is advantageous in formulating a dome model after cracking. The meridians have pure compression forces, but hoops have compression forces in the dome’s top part and tension forces in the lower part. The meridian compression stress improves the masonry’s tensile strength and opposes the cracks widening. When the hoop stresses reach the masonry tensile strength in a dome, cracks occur and develop along the meridians; thus, the dome behavior gradually changes from a rigid shell stiffened by hoop forces toward a dome divided by meridian cracks. Furthermore, dynamic actions, earthquakes, environmental vibrations, and differential foundation settlements widen the cracks.

In most cases, dome or vault intrados and extrados cracks begin several decades after constructing the domes. This delay in cracking indicates that domes or vaults behave as solid and membrane structures supporting tensile stresses in the early ages of their construction. After cracking, tensile hoop forces disappear in the dome’s lower rings, and meridian cracks expand upwards toward the center until a new static equilibrium configuration is attained. Finally, the cracks reach a height that is the theoretical location of the beginning of the tensile zone in the hoops. After cracking, the dome behaves as a set of arches, and full semicircular arches are assumed, although the top part of the dome turns into an uncracked cap.

The cylindrical shells of barrel vaults with zero Gaussian curvature in one direction do not take advantage of shape strength in two orthogonal directions, unlike spherical shells, and are probably more prone to cracking and deformation. Barrel vaults supported at their boundaries are the simplest form of arched roof sheltering spaces by a rectangular plan in Romanesque and Roman architecture. The masonry barrel vaults at cracking state diminish to a series of parallel or side-by-side arches buttressed by lateral walls or tie rods. The sliced vault’s Heyman model represents the cracking cross vault’s resisting structure in which webs are separated and sliced into several arches with different spans supported by diagonal ribs. The thrust caused the following deformation of the vault’s piers, with a small span widening and increased cracking. The sliced vault’s reaction to this settlement produces a minimum thrust state.

**Figure 5:**
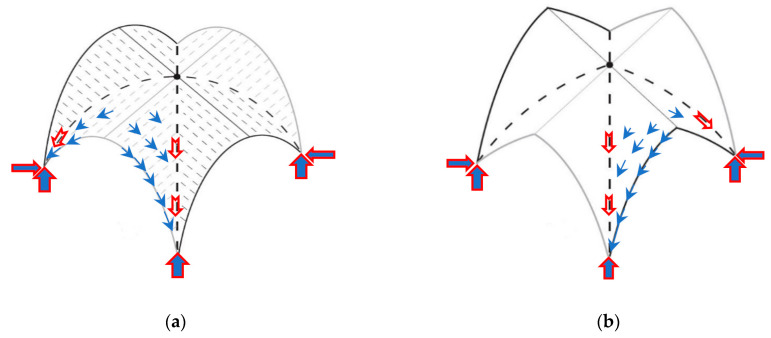
Force transmission from the vault to supporting columns (piers) for (**a**) groin vault, (**b**) rib vault.

The vault stress state may be analyzed using shell membrane solutions in tensile strength and before cracking. Thus, the masonry barrel vault’s static analysis is diminished to a masonry arch. In most Romanesque and Gothic-style churches, the central barrel vault sidewalls are lightened and covered using a series of small chapels, lateral aisles, or flying buttresses to carry the main vault’s thrust. Depending on the vault’s mass distribution and geometry, various failure mechanisms may occur, e.g., local, semi-global, or global. Local mechanism happens entirely inside the vault, the semi-global includes both the vault and a side wall, while global failure, which is relatively uncommon, assumes the collapse of the vault with the sidewalls. The groin vault is produced by the intersection of two cylindrical barrel vaults and two intersecting semicircular arcs highlighted with dashed curves in Figure 5a. A rib vault is formed if a curved surface replaces the cylindrical cross-sections with pointed arcs (Figure 5b). The rib vault is lighter and structurally safer than the more massive barrel/groin vaults. The rib’s points of convergence determine the placement of the supporting vertical columns in which the vault thrusts are concentrated and should be countered by flying buttresses, tie rods, etc.

## 4. Crack Patterns and Strengthening of Masonry Structures

Common crack locations and patterns observed in masonry buildings are (1) in-plane diagonal or shear cracks, (2) out-of-plane partial or total collapse, (3) cracking near openings, (4) separation between roof and walls or wall intersections, and (5) cracking in the arch and vault. The strength of URM walls subject to in-plane actions depends on several failure modes: joint sliding and diagonal tension, which are shear-controlled, and toe-crushing, which is flexure-controlled. Generally, rocking or sliding governs the response for URM columns with low vertical axial pressure and diagonal tension, and toe-crushing force-controlled actions are more frequent in high vertical axial stress. Due to height and sensitivity to winds, earthquakes, and environmental vibrations, surveying cracking patterns is challenging for some structures, e.g., bell towers, the Garisenda leaning tower in Bologna, and the leaning tower of Pisa. The vibrations induced by the bells and nearby vehicles may be a source of damage through cracks, and monitoring should frequently be performed on such buildings. So, dynamical structural identification procedures are utilized to measure structure frequencies, oscillation modes, and dynamical features. Then, different measurements are compared over time, enabling continuous monitoring of any damage development.

**Figure 6:**
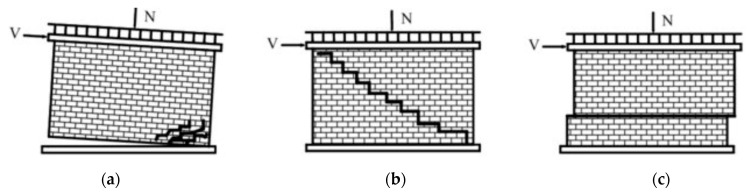
Crack patterns in UnReinforced Masonry (URM) walls: (**a**) rocking failure; (**b**) diagonal cracking; (**c**) bed-joint sliding [7].

Another approach is employing Fracture Mechanics (FM), which studies structural integrity and behavior. In 1989, Bocca and Carpentieri [53] determined, for the first time, Fracture energy, G_F_, and the critical value of the stress-intensity factor, K_IC_, for brick masonry specimens tested in bending with different notch depths, and the experimental results are compared with numerical simulations, obtained through a cohesive crack model developed originally for concrete. In 2022, Greco et al. [54] determined crack propagation analysis in masonry structures via an interelement cohesive fracture approach: the assessment of mesh dependency issues. Several clear patterns were confirmed and detailed (Figure 6), and the patterns are similar to experimental results from civil engineering [7].

### 4.1. Masonry Walls

According to ASCE 41-17 section C11.3.3.3 [37], the URM with specific wall height-to-thickness (H/t) provides significant resistance by arching actions to the out-of-plane forces and does not require strengthening. The arching action is developed in a masonry wall with H/t < 8 if constructed among stiff supports. The wall arching action is neglected in URM thin walls subjected to out-of-plane loading, and strengthening is required for H/t > 20, in which large deflections may lead to partial or total collapse. The airbag tests determine masonry walls’ out-of-plane strength, deformation capacity, and arching properties [55], considering connections and edge details standardized by ASTM [56]. The dynamic out-of-plane stability of URM/RM walls may be evaluated using a shake table test and realistic boundary conditions [57]. If not well connected to perimeter supports, masonry walls cannot develop arching action. They should be restrained on each side for out-of-plane forces by anchoring steel plates [58].

High compression stresses at lower levels of irregular masonry walls can induce out-of-plumbing and produce orthogonal actions to the wall plane, leading to local failures. The vertical walls vary significantly in thickness, and their eccentricity can lead to walls bulging outwards and cracks, especially near window openings. A traditional restoration includes using steel ties at different levels to rehabilitate the wall’s connections. Masonry wall/foundation inclination and rotation also arise with nonuniform soil settlement, a problem for wall statics resting on deformable soils, e.g., clay or silt. Furthermore, soil creep deformations may lead to gradually increasing wall tilting. Time-dependent loads, height variations of the water table, or environmental conditions can lead to wall failure in such cases.

The RM wall shear capacity can be estimated by employing the strut-and-tie model (truss) analogy equilibrium, which assumes that the strengthened panel’s in-plane shear strength depends on the strengthening scheme and masonry substrate contributions (see Figure 6). The externally bonded vertical fabric stipes improve the in-plane flexural failure modes, e.g., rocking, toe-crushing, and activating the strut-and-tie resisting mechanism. Diagonal cracking is formed in the shear-controlled failure modes for walls under heavy vertical load, so horizontal or diagonal fabric stripes enhance wall shear strength. The masonry substrate contribution may be assumed to equal the in-plane shear strength of URM.

The strengthening scheme contribution depends on the fiber tensile capacity and anchorage length. The connection between the substrate and the reinforcing system is enhanced by applying specific connectors inserted inside the masonry before the mortar’s final covering layer is implemented. The bed joint reinforcement is typically used to improve the wall shear capacity. Moreover, the composite materials are applied to both wall sides, covering the entire surface or vertical/horizontal discrete strips with transverse connectors for multi-leaf/wythe walls. Another possible option is grout injection. In this approach, masonry cracks are filled through a proper pattern of drilled holes to enhance the continuity of wythe (multi-leaf) wall sections [59].

**Figure 7:**
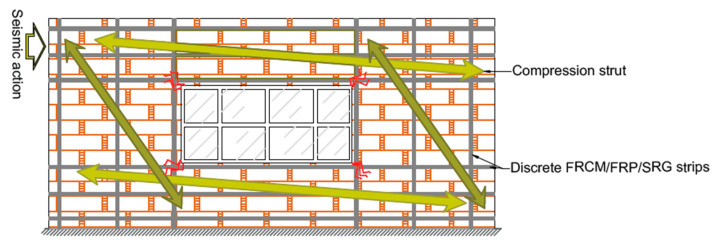
Strut-and-tie-model for the in-plane reinforcement of masonry walls with opening.

**Figure 8:**
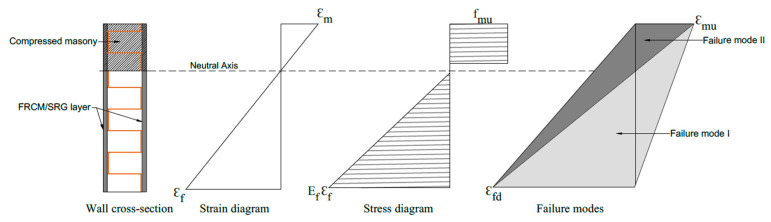
Cross-section of the wall, strain, stress profiles, and failure modes.

The RM wall in-plane flexural strength is correlated with the following governing failure modes: (1) crushing the masonry in compression represented by stress block and neutral axis depth is calculated for the fabrics in tension and masonry in compression; (2) masonry substrate debonding, fabric-matrix interface debonding, fabric slippage inside the matrix, or tensile fabric rupture, which is masonry, remains in the elastic range in compression (Figure 7). The externally bonded steel fabric is assumed to be elastoplastic, and the strain profile is calculated based on the plane-sections-remain-plane hypothesis. The stress and strain profile and failure modes are represented in Figure 8, almost the same as RC behavior [60]. Applying FRP bars or strips on wall surfaces can avoid both in-plain and out-of-plain collapse mechanisms. The explanations for notations represented in Figure 8 are as follows: ε_m_ is the compressive strain of masonry, ε_f_ is the tensile strain of FRP/FRCM/SRG, ε_mu_ is the ultimate compressive strain of masonry, ε_fd_ is the design tensile strain of FRP/FRCM/SRG, E_f_ is the stiffness (mean value) of FRP/FRCM/SRG provided by tensile tests, and f_mu_ is the compressive strength of masonry.

**Figure 9:**
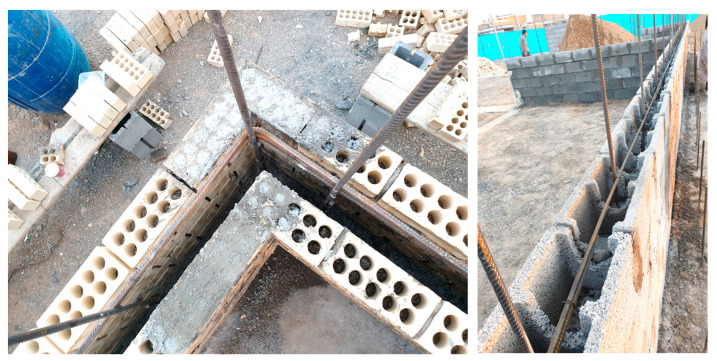
RM walls reinforced with steel bars, Shiraz, Iran.

Eurocode 6 applies to the design of unreinforced, reinforced, prestressed, and confined masonry buildings. According to EN 1996-1-1 [61], when analyzing RM walls subjected to vertical loading, allowance in the design should be made for vertical loads directly applied to the wall considering eccentricities and second-order effects. Reinforcement is added to RM walls to provide ductility and strength and improve serviceability. Bars or mesh are embedded in mortar or concrete so that all wall materials act together in resisting action effects. Figure 9 shows a single-leaf wall consisting of a longitudinal joint filled with mortar and tied with horizontal and vertical steel bars or bed joint reinforcement, resulting in homogeneous action under loads. Reinforced masonry members shall not crack unacceptably or deflect excessively under serviceability loading conditions.

### 4.2. Masonry Archs and Vaults

**Figure 10:**
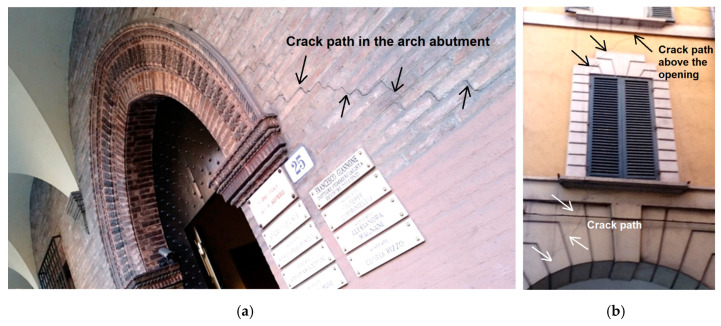
(**a**) Crack path in the arch abutment due to thrust pressure; (**b**) crack near window openings, Bologna, Italy.

Masonry domes, barrel, cross, and cloister vaults, have two stress states under loading: the primary phase without cracking and an ultimate cracked phase where the tensile strength vanishes. The membrane solution reasonably estimated the initial state to sustain tensile stresses, and after cracking, the ultimate state is modeled in the rigid no-tension framework. The vault stress state remains constant for a long time if loading is relatively low and if the masonry provides adequate tensile resistance. The cross vault’s transition from uncracked to cracked state is similar to masonry domes. The cracked cross vault webs are separated into some series of arches with different spans supported by the diagonal ribs, and thrust causes the vault’s abutment deformation, span broadening, and increased cracking. The arch’s horizontal thrust pushes the supports outward, leading to crack formation at extrados abutments, as illustrated in Figure 10a. Due to high compression forces (horizontal thrust), cracks appear in the arch abutments - Figure 10b shows where cracks appear above the openings. The diaphragms’ structural performance of existing masonry buildings is crucial since they transfer horizontal forces to the walls mainly when seismic/wind loads are engaged.

Therefore, avoiding excessive mass increase at the floor level is vital to improve existing floors’ in-plane stiffness and redistribute horizontal forces. Additionally, wall-to-diaphragm connections are essential to out-of-plane stability since inadequately attached walls to top diaphragms are less stable and behave in cantilever mode. Likewise, connections among floors, roofs, walls, or wall-to-diaphragms are essential for suitable box-like responses under seismic forces. This is conducted by steel tie rods, timber beams, and composite strips acting as ties. Using composite material strips or diagonal metallic belts fixed at the extrados (outer surface) of wood, masonry floors, or arcs can improve the diaphragmatic action [62].

**Figure 11:**
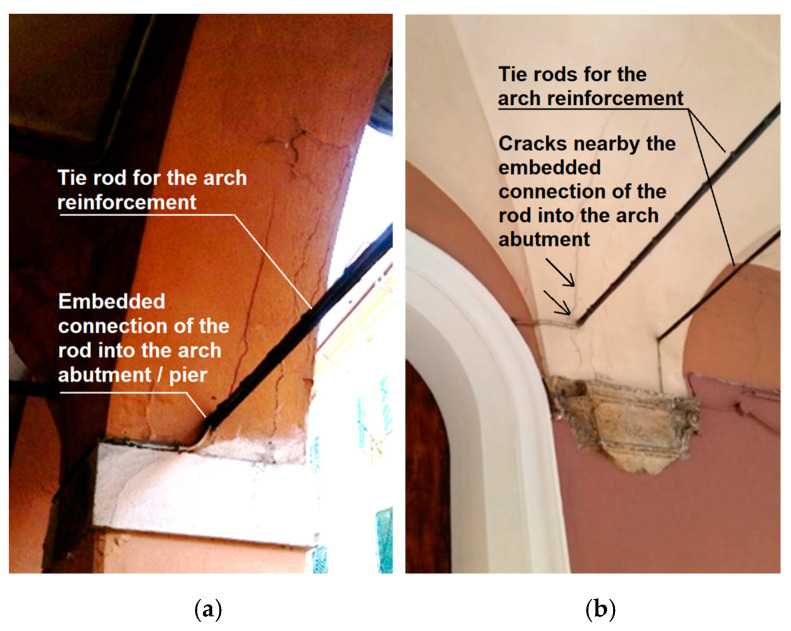
Single (**a**) and double (**b**) steel tie-rods used for arch and vault reinforcement, Bologna, Italy.

Increasing arch or vault thickness is another traditional strengthening solution by installing RC slabs on the extrados. Steel or timber tie-rods are mainly used to support the horizontal thrust of arches, vaults, and roofs. They are particularly suitable for better connecting structural elements, e.g., masonry walls and floors. The combined use of horizontal and vertical tie-rods increases the entire structure’s resistance. This can increase the mass without the possibility of removing it. Another traditional method consists of inserting tie-rods generally to equilibrate the thrust forces (see Figure 11), but these require additional maintenance against corrosion and rusting. This is achieved by utilizing a suitable coating or zinc galvanization.

**Figure 12:**
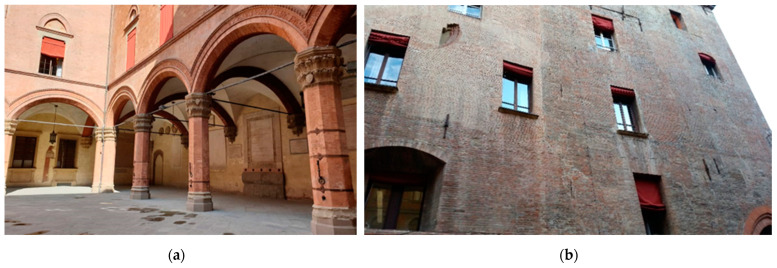
(**a**) A coupled system of cross vaults reinforced by steel tie rods; (**b**) reinforcement by steel tie-rod and bar-shaped anchor plate, Bologna, Italy.

Figure 12a shows a coupled cross vault system in Bologna, Italy, in which the central columns mainly support gravity forces since tie rods are applied for transmitted thrust action. Figure 12b shows arches/vaults strengthened with steel tie systems passing through the floors and piers with anchor plates attached to their heads. Tie-rods provide an efficient joint connecting the main components of the structure, e.g., walls and floors, and control arches and vaults’ horizontal thrusts due to dead, live, and seismic loadings. Furthermore, such elements may increase the horizontal displacement and ductility capacity of arched and vaulted structures and improve seismic response. Structural ductility is the capacity to sustain strength during failure mechanism development.

The steel ties can withstand only tension stresses, while masonry materials provide compressive strength. Tying opposite walls or confining rings improves the structures’ box-like performance, providing effective connections between masonry walls. The failure mechanism of masonry structures reinforced by elastoplastic systems, such as steel tie rods, is more ductile since steel ties are in a plastic state with constant internal stresses. The tie-rod and anchor plate brace the masonry walls, arcs, and vaults against lateral displacements and spread apart. An anchor plate is a large plate attached to the end of a tie-rod or bolt with different sizes and thicknesses. Due to visibility on the exterior side of a brick wall, many anchor plates are manufactured in a decorative style such as an x, star, or bar shape [63].

**Figure 13:**
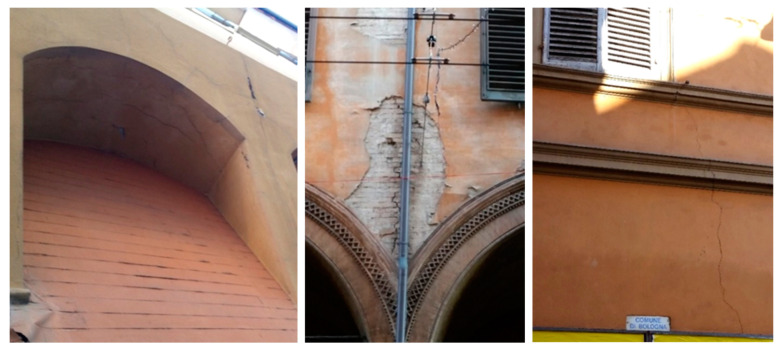
Typical cracking patterns due to gravity loads and lack of tension resistance and load distribution, Bologna, Italy.

Gravity loads are the most long-lasting actions on masonry buildings and are accountable for several damages and crack patterns. Additionally, an internal resistance occurred in the masonry body depending on the structural element’s geometry (see Figure 13a). The principal consequences of assuming masonry materials cannot resist tension are that the masonry is incompatible with load scattering or loads not diffusing within a masonry continuum. Figure 13b shows a vertical crack above a column at the intersection of two cross vaults. Figure 13c illustrates a typical cracking pattern on the perimeter walls, especially near window openings. Due to load dispersion deficiency in masonry walls, these cracks may originate at the window’s corners and grow vertically to the top and bottom. Cracks may open, but sliding cannot occur; therefore, crack openings should be widened and filled with liquid mortar. Vertical cracks may also develop at the intersection of walls. Inclined cracks by about 45° can be observed if the settlement occurs or the foundation’s central portion subsidence, producing shear stresses on the masonry.

**Figure 14:**
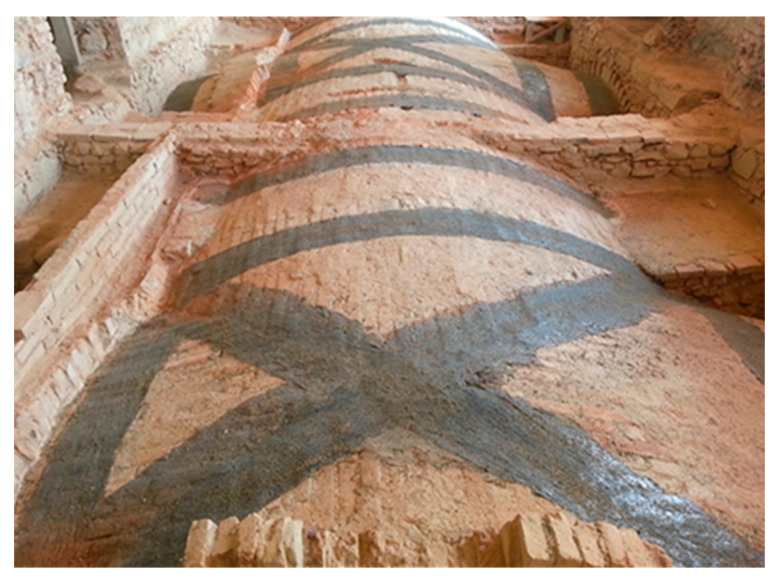
Vaults strengthening at the extrados [64].

Composite reinforcing systems can be installed all over the surface of the arch or vault extrados/intrados or implemented in discrete stripes. Although the extrados stripes installation needs floor and backfill removal, requiring more time and cost, it preserves intrados decorative paintings and provides a better connection between the substrate and the composite due to curvature. After the substrate preparation and cleaning, the first mortar layer is spread, and the fabric strip is placed by pushing on the mortar until the mortar comes out of the fibers spaces. Then, the second mortar layer is spread to cover the fabric strip adequately, and finally, a transverse fabric strip with primer and mortar is implemented to complete the reinforcing system. The strengthening application on the extrados of the arch/vault is a very efficient solution since no hinges are allowed to form on the strengthened surface side. Hence, the new collapse mechanism will activate with a greater load multiplier than the un-strengthened one. Figure 14 shows the reinforcement of the masonry vault with extrados composite strips [64].

Garmendia et al. [65] proposed a strengthened solution for barrel brick masonry vaults in the laboratory with SRG/FRCM with various fibers covering the entire intrados/extrados surface and tested it under cyclic vertical loads applied symmetrically at each span quarter. The results reveal a failure mode change from brittle to ductile, and specimens strengthened only at the extrados side had the best displacement capacity. Installing composite materials at the extrados/intrados led to the activation collapse mechanisms with higher hinges and critical loads estimated by plastic Limit Analysis methods. The ultimate strength was improved approximately ten times more than the un-strengthened specimens. If both intrados and extrados surfaces were strengthened, tests showed the highest ultimate capacity than extrados strengthening, and the failure mode was voussoir crushing.

Different fabric and mortar designs have different characteristic responses that correlate with cracking spacing and composite stiffness [66]. Microstructural evaluation refers to various instruments used to investigate FRCM/SRG failure modes and crack spacing and formation to evaluate the interaction of yarns with matrix in the crack opening, fiber debonding, and fiber fracture [67].

### 4.3. Masonry Columns (Piers)

In URM columns stability analysis, the column is assumed to be hinged at both ends due to the constraining impacts of floors and buttresses, and the elastic strains change linearly with distance across the cross-sections from the neutral axis. If the axial compression force is applied inside the central core of the column cross-section, all the points become compression.

The columns’ eccentricity is the distance of the stress distribution resultant from the cross-section centroid, and eccentrically loaded masonry piers act variously from RC pillars. In URM columns, cracks propagate everywhere in sections and significantly reduce the strength, though in RC pillars, concrete within adjacent cracks and rebars sustains the tensile stresses. In addition, elastic flexural deformations develop axial load eccentricity and narrow the column section’s resistant areas. The mortar shrinkage deformations and changing external column or wall thickness with the height affect the stress distribution and significantly boost the destabilizing impacts of axial load eccentricity [68].

The strengthening schemes with composite or steel materials are commonly applied to masonry structural elements using vertical/horizontal stripes or continuous full coverage. The reinforcement layout choice depends on the cross-section features, fabric layers, geometric and mechanical features, and masonry substrate. Using a discrete strip configuration, the strengthening system is applied to entire element surfaces made of soft limestone brick to clay brick or hard stones. FRCM/SRG systems are used for jacketing masonry columns without adding considerable mass that amplifies the seismic demand. These systems provide confinement effects for masonry columns and increase axial strength and ductility deformation capacity. The research on masonry columns wrapped with composite materials indicates that axial capacity and vertical and lateral displacements might increase by up to 200 percent concerning unconfined piers. The number of fabric layers improves the confinement effectiveness and modifies the column failure mode [60]. The confining action in columns strengthened with FRCM/SRG is affected by matrix and fabric characteristics. Applying a high-strength fabric embedded in a weak mortar matrix may reduce the confining effectiveness due to early mortar cracking [69].

Using discontinuous fiber stripes or steel wrapping, the masonry piers confinement is a powerful technique for increasing the compressive strength based on the lateral deformation limitation and is especially effective on circular cross-section elements. The compressive strength increase usually depends on the provided confining pressure, the confining system’s stiffness and strength, the column cross-section’s shape, and masonry mechanical properties [70].

**Figure 15:**
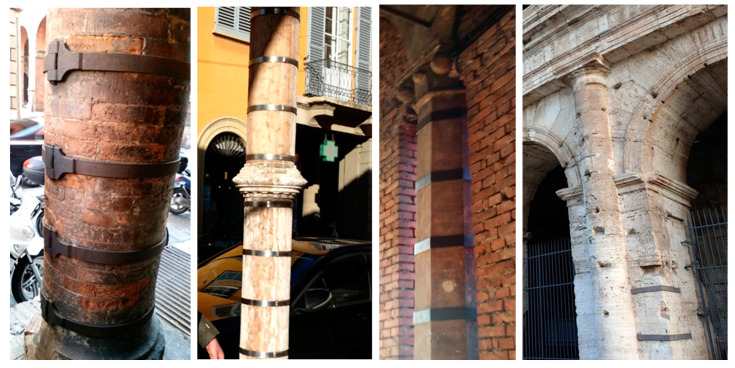
Brick and stone columns confined with discontinuous wrap configuration, Bologna, Italy.

An example of brick and stone masonry column confinement with a discontinuous wrap configuration is presented in Figure 15, in which strengthening intervention of circular, rectangular, and hexagonal columns was conducted using a discrete steel stripes layout.

SRG/FRCM techniques also designated an alternative strategy to strengthen masonry columns/piers [71]. These systems increase the axial strength, ductility, and deformation capacity of masonry piers with their confinement act, necessary for conservation and restoration requirements, without a significant mass increase (that increases seismic force) and with minimum stiffness increase [72].

The FRCM/SRG qualification Is established on a test procedure that provides data on material properties, force, and deformation limit states, including failure modes, according to specified test approaches [73,74]. The qualification parameters should be representative only of the procedure that experienced the experimental tests and cannot be developed for diverse scenarios. Empirical results are sensitive to fabric properties and architecture, matrix characteristics, the interaction between matrix and fabric, and the number of fabric layers embedded within the FRCM/SRG composite and affect the FRCM/SRG parameters [75,76,77,78,79].

## 5. Conclusions

The demand for safety assessments and restoration of masonry constructions is increasing due to high vulnerability under subsidence, gravity, and seismic actions that require appropriate strengthening measures. A comprehensive interdisciplinary analysis can diagnose masonry buildings based on technological surveys, non-destructive tests (NDTs), destructive tests (DTs), and the interpretation of crack patterns.

This study overviews typical crack patterns developed in masonry structural elements that may provide helpful information regarding the collapse mechanisms that allow for reliable retrofitting interventions.

The application of traditional and modern techniques for repairing and strengthening masonry walls, arches, vaults, domes, and columns has been presented, e.g., externally bonded fabric-reinforced cementitious matrix (FRCM), steel-reinforced grout (SRG), and tie-rod systems. Composite reinforcing systems significantly improve masonry structural elements’ behavior, such as load-bearing and displacement capacity, crack propagation, compatibility with the masonry substrate’s physical and mechanical properties together, with corrosion resistance, with the benefit of low weight and the possibility of removal. Tie-rods connect the main structure components, e.g., walls and floor, and control arches and vaults horizontal thrusts due to dead, live, and seismic loadings. Although they are prone to corrosion, such elements may increase the horizontal displacement and ductility capacity of arched and vaulted structures and improve seismic response. Limit Analysis’s static and kinematic theorems help determine failure mechanisms and associated critical loads within the framework of the rigid no-tension model.

In addition, several research results in automatic surface crack detection for URM walls are presented, considering crack detection based on machine learning and deep learning algorithms.

The manuscript sets a practical perspective, providing an inclusive list of papers describing the most important latest research in this field; thus, the paper is helpful for researchers and practitioners in the masonry structures field.
